# Real-time decentralized model predictive control for cooperative multi-robot object transport: experimental validation

**DOI:** 10.1038/s41598-026-41881-w

**Published:** 2026-03-22

**Authors:** Ibrahim Muhammed, Ayman A. Nada, Haitham El-Hussieny

**Affiliations:** Department of Mechatronics and Robotics Engineering, Egypt-Japan University of Science and Technology, Alexandria, Egypt

**Keywords:** Adaptive control, Constraint handling, Cooperative object transportation, Decentralized model predictive control (MPC), Multi-robot systems, Real-time control, Trajectory tracking, Engineering, Mathematics and computing

## Abstract

This paper presents an experimental validation of a decentralized Model Predictive Control (MPC) framework for cooperative object transportation utilizing a multi-robot system consisting of two mobile robots. Each robot is a differential-drive robot that independently solves local constrained optimization problems while ensuring global coordination through joint-space coupling. The formulation explicitly captures nonlinear kinematics, revolute-prismatic joint dynamics, inter-robot constraints, and dynamic obstacle avoidance within a real-time optimization setting. Adaptive weighting of cost terms is employed to balance trajectory tracking and formation objectives under varying task demands. The framework is deployed on a physical testbed integrating vision-based pose estimation, sensor fusion via a Kalman filter, and a ROS 2 control infrastructure. Experiments across point-to-point, curvilinear, and obstacle-rich scenarios show accurate trajectory tracking, strict constraint satisfaction, and robustness to environmental uncertainties. These results substantiate decentralized constrained MPC with adaptive weights as a practical and scalable solution for real-time multi-robot cooperative transport along arbitrary reference paths.

## Introduction

The increasing demand for automation in domains such as logistics, manufacturing, and search-and-rescue has driven the development of cooperative multi-robot systems capable of executing complex manipulation and transportation tasks. Among these, cooperative object transport poses significant challenges due to the need for precise coordination, the presence of nonlinear and constrained dynamics, real-time decision-making requirements, and robustness to environmental uncertainties^1–3^.

Model Predictive Control (MPC) has emerged as a powerful framework for addressing such challenges, offering the ability to predict future states, enforce state and input constraints, and optimize performance over a finite horizon^4–7^. In multi-robot contexts, decentralized implementations of MPC are particularly attractive, as they allow agents to compute local control actions autonomously while maintaining coordination through limited communication. This improves scalability, fault tolerance, and adaptability in dynamic environments^8–10^.

This paper presents a decentralized MPC framework for cooperative object transportation, formulated for differential-drive mobile robots physically coupled via revolute-prismatic joints. The proposed control architecture explicitly incorporates nonlinear kinematics, inter-agent constraints, collision avoidance, and trajectory tracking objectives. Coordination is achieved through an event-triggered communication strategy that reduces bandwidth usage while ensuring synchronization.

In addition to the control design, we develop and experimentally validate a real-time implementation using two TurtleBot3 Burger platforms in a constrained indoor environment. The system integrates ROS 2-based decentralized controllers, vision-based pose estimation, and Kalman-filter-based sensor fusion. A series of experiments evaluates the framework’s ability to achieve trajectory fidelity, enforce physical constraints, and maintain robustness under uncertainties such as sensor noise and dynamic interactions.

By bridging theory and practice, this work demonstrates the feasibility of deploying decentralized constrained MPC in real-world cooperative robotic systems. It contributes to the growing body of research on distributed control for multi-agent systems, offering a practical architecture for scalable and resilient implementation in dynamic and constrained environments.

## Literature review

Cooperative object transportation using multi-robot systems has been extensively investigated due to its broad range of applications in industrial automation, logistics, disaster response, and defense. Control strategies in this field typically fall into centralized or decentralized paradigms, each with distinct trade-offs in scalability, robustness, and computational overhead.

Early research predominantly favored centralized control^11^, where global coordination is achieved via complete state sharing among agents. For instance, Drew^12^ explored centralized multi-agent architectures for search-and-rescue missions, achieving high coordination fidelity in structured environments but suffering from poor scalability and fragility to communication loss. Similarly, Kvalsund et al.^13^ demonstrated centralized control of modular robots in controlled lab settings, though performance degraded in unpredictable scenarios due to centralized dependencies and computational bottlenecks.

Decentralized approaches emerged to mitigate these limitations, enabling agents to make local decisions based on partial observations. Wang et al.^14^ presented an early decentralized object transport scheme using implicit coordination. De Sousa et al.^15^ later offered a comprehensive analysis of decentralized multi-agent systems, emphasizing their resilience and adaptability in dynamic environments.

Among decentralized strategies, Model Predictive Control (MPC) has gained attention for its ability to optimize future actions while respecting input and state constraints. MPC’s predictive and constraint-aware formulation makes it suitable for multi-robot coordination under dynamic and uncertain conditions. García et al.^4^ established its theoretical foundations, which remain widely adopted. Mayne et al.^16^ further advanced the field by developing tube-based formulations that guarantee recursive feasibility and robust constraint satisfaction in nonlinear systems. This has influenced a wide range of distributed and robust MPC methods.

Recent works have applied these principles to cooperative transportation tasks. Kennel-Maushart and Coros^2^ developed a payload-aware MPC framework for non-holonomic robots with stability-aware trajectory planning. Nikou and Dimarogonas^17^ extended this with a decentralized tube-based MPC strategy for multi-agent systems under uncertainty, offering provable guarantees on safety and performance-elements crucial for real-world deployment.

Another key advancement in recent years has been the improvement of computational efficiency for real-time embedded MPC. Fiedler et al.^5^ evaluated solvers like IPOPT and ACADOS for real-time control, demonstrating significant reductions in latency. Wu et al.^18^ tackled the challenge of handling non-convex constraints through iterative distributed MPC, offering a scalable approach for tightly coupled robot-object systems.

In parallel, attention has turned to communication efficiency. Event-triggered MPC schemes have been proposed to limit bandwidth consumption while maintaining stability. Hu et al.^19^ and Wang et al.^20^ introduced event-triggered asynchronous distributed MPC with provable stability and Zeno-free guarantees, offering compelling frameworks for resource-constrained multi-robot systems-relevant to our event-based communication strategy.

Complementing these algorithmic contributions, vision-based decentralized MPC frameworks have also emerged. Guan^8^ employed distributed MPC with visual feedback for formation tracking, improving synchronization under controlled lighting but facing robustness issues in dynamic environments. Matsunaga et al.^10^ and Huang and Zhang^9^ introduced hardware-specific solutions (suction mechanisms and ball-string-ball linkages, respectively), with trade-offs in generality and implementation flexibility.

Integrated control-planning frameworks are another active research area. Jaafar et al.^1^ proposed a joint planning and MPC scheme to reduce coordination latency in multi-robot transport, though its scalability was hindered by computational demand in dynamic environments.

Although adaptive or gain-scheduled weighting has been explored in several MPC contexts-including adaptive tuning for linear systems, robust disturbance rejection, and performance shaping^4,16^-these formulations differ substantially from the decentralized and physically coupled setting considered here. Existing adaptive MPC schemes typically rely on centralized optimization, simplified or linearized dynamics, or holonomic robotic platforms, and therefore do not address the requirements of cooperative payload transport, where nonholonomic constraints, inter-agent geometry, and coupling forces must be handled consistently.

Despite significant progress in distributed MPC and cooperative manipulation, existing approaches rarely address the combined challenges of real-time decentralized control, physical coupling, and uncertain sensing inherent to cooperative payload transport. Most prior work relies on simulations or simplified laboratory setups^17,18^, often assuming reliable communication, idealized dynamics, or synchronous state sharing. In contrast, the approach proposed in this work integrates (i) decentralized NMPC for nonholonomic mobile robots mechanically coupled through revolute-prismatic joints, (ii) delay-aware peer-state estimation within an event-triggered communication framework, and (iii) curvature- and speed-dependent log-domain weight adaptation tailored for embedded real-time execution. This combination enables online modulation of tracking and formation priorities while preserving feasibility and smooth control actions. To the best of our knowledge, no prior study has experimentally validated an adaptive-weight decentralized NMPC framework on physically coupled robots operating under obstacle-rich, noise-prone, and asynchronously updated sensing environments. By addressing these practical challenges through a hardware-based implementation with vision–odometry fusion and real-time optimization, this work contributes a validated and scalable solution for cooperative multi-robot object transport in constrained environments.

## System description

This section details the experimental hardware and software infrastructure used to validate the proposed decentralized MPC framework for cooperative object transport. The system comprises two differential-drive mobile robots, a custom-designed passive payload, a vision-based pose estimation system, and a ROS 2-based distributed control architecture.

### Robotic platform

Two **TurtleBot3 Burger** platforms were used due to their modularity, affordability, and seamless integration with ROS 2^21^. Each robot employs a differential-drive configuration using two Dynamixel XM430-W210 servomotors, controlled via an onboard OpenCR controller^22^. Integrated sensors, including wheel encoders and an IMU, enable real-time feedback for velocity and heading estimation.

**Fig. 1:**
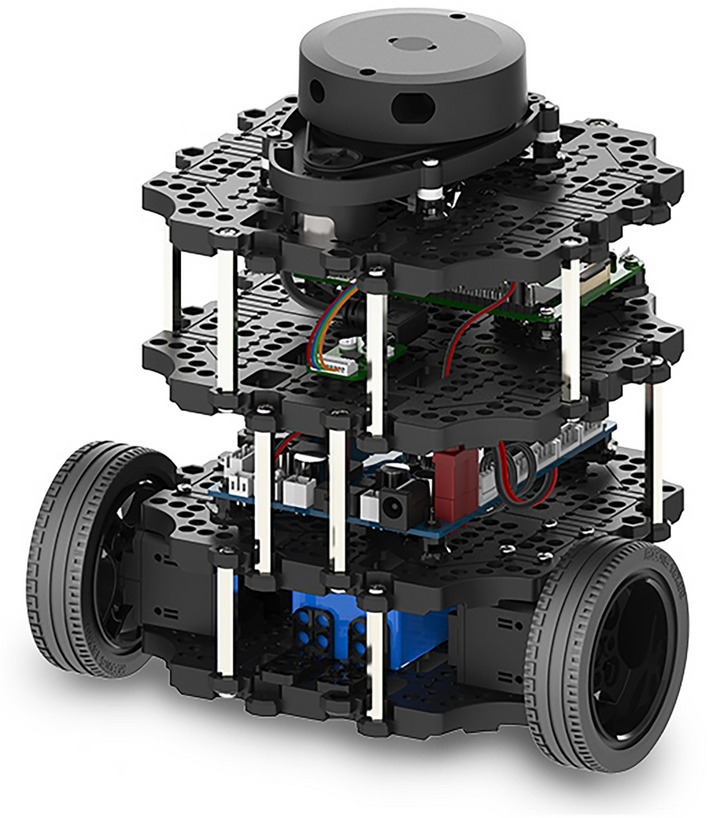
TurtleBot3 Burger platform used for decentralized cooperative transportation.

Robots execute velocity commands via the cmd_vel ROS topic at $10\,\text {Hz}$, and publish odometry at the same rate. The wheel radius is $0.033\,\text {m}$, and the track width is $0.16\,\text {m}$, consistent with the nonholonomic kinematic model described in “Decentralized nonlinear MPC”. The robot platform is illustrated in Fig. 1.

### Payload configuration

A lightweight acrylic payload was designed to simulate cooperatively carried objects. It is mechanically coupled to the robots using custom-built revolute-prismatic (RP) joints, allowing both angular and translational compliance. This configuration replicates realistic object manipulation dynamics while remaining structurally simple.

**Fig. 2:**
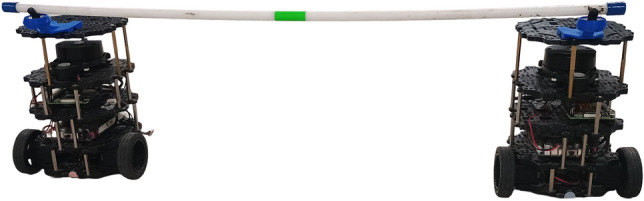
Dual robot configuration with revolute-prismatic coupling. Right: mechanical zoom of 3D-printed joint.

The prismatic link permits extensions between $0.12\,\text {m}$ and $0.32\,\text {m}$, while the revolute joint supports angular deviations up to $\pm 360^\circ$. The joint states are inferred from robot poses and geometric linkage constraints, eliminating the need for embedded joint sensors. The dual-robot configuration and joint details are shown in Fig. 2.

### Experimental workspace

Cooperative transportation tasks were executed in a $3 \times 3\,\text {m}^2$ indoor lab environment featuring a smooth vinyl surface to minimize wheel slippage. Workspace boundaries were defined using colored tape and four AprilTag markers (tag36h11 family)^23^, which are placed at known world coordinates to compute a homography transformation for global localization.

**Fig. 3:**
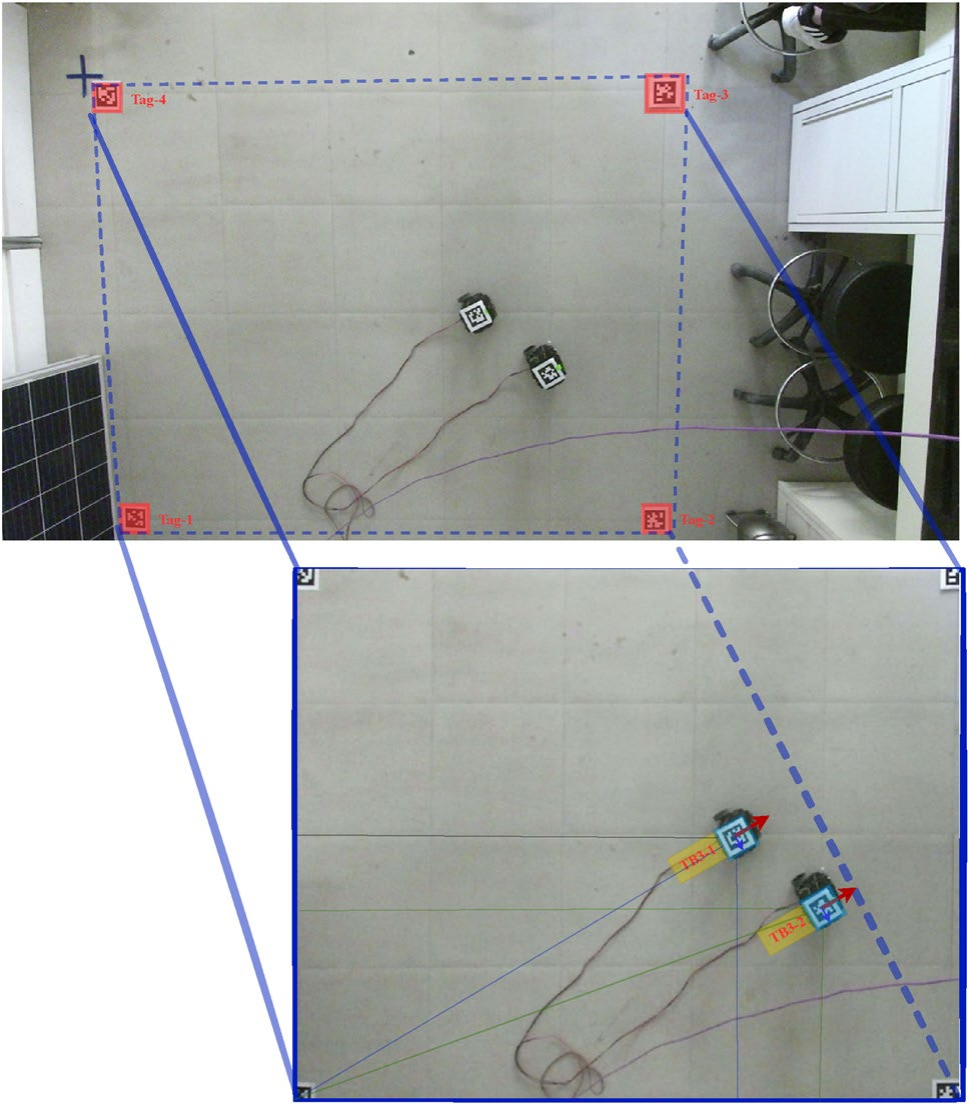
Top view of experimental workspace with AprilTags and boundary overlays.

A Microsoft Kinect V2 RGB-D camera^24^ was mounted $2.5\,\text {m}$ above the workspace, streaming $1920 \times 1080$ RGB images at $30\,\text {Hz}$. AprilTags affixed to each robot and the payload allow real-time 6-DOF pose tracking with sub-centimeter positional and $2^\circ$ angular accuracy. A top-down view of the workspace is shown in Fig. 3.

### Pose estimation and filtering

AprilTag detections are mapped to world coordinates using the computed homography matrix. However, vision-based measurements are prone to latency and temporary occlusions. To mitigate this, an Extended Kalman Filter (EKF)^25^ fuses global pose estimates from vision with local odometry to produce reliable, low-latency position feedback.

**Fig. 4:**
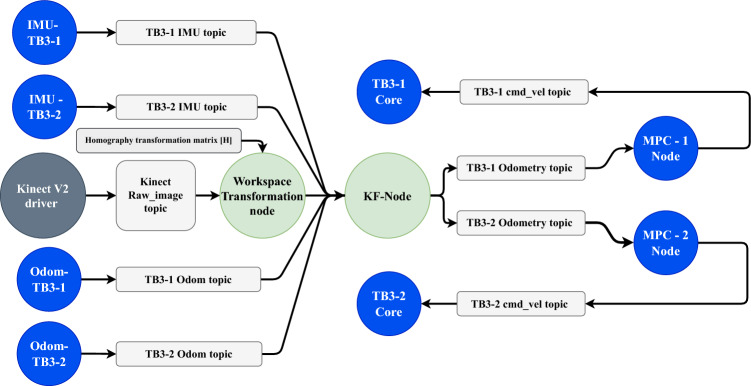
System architecture showing Kinect-based pose estimation, state transformation, and decentralized MPC execution.

The EKF is implemented as a standalone ROS 2 node and publishes fused pose estimates on the /KF_Odom topic, which are subsequently consumed by each MPC node for closed-loop feedback control. The sensor fusion pipeline is illustrated in Fig. 4.

### ROS-based control and communication

The system architecture is built entirely within the ROS 2 Humble middleware^26^, leveraging the Data Distribution Service (DDS) for real-time communication. Each robot runs its own MPC node as a separate process, performing real-time optimization using the acados solver^27^.

A lightweight event-triggered communication strategy is implemented to reduce bandwidth usage while maintaining coordination. Robots broadcast updated state information only when joint stretch or relative position deviation exceeds specified thresholds. DDS QoS settings are configured for reliable and deterministic delivery. The full ROS-based system architecture is shown in Fig. 4.

This architecture supports scalability to additional agents and sensors, and is compatible with advanced perception pipelines such as LiDAR- or SLAM-based localization.

## Pose estimation and integration

Accurate real-time feedback is essential for cooperative transport. We adopt a hierarchical fusion pipeline that combines camera, IMU, and encoder odometry, ensuring robust pose estimation even under partial sensor loss. The pipeline consists of three stages: (i) camera-based global pose (if available), (ii) IMU- and odometry-based local tracking, and (iii) Kalman filter fusion.

### Stage 1: camera-based pose estimation (if available)

An overhead Kinect V2 camera with fiducial markers provides global object pose when available. The image-plane coordinates $$\begin{bmatrix} u&v \end{bmatrix}^\top$$ are mapped to world-plane coordinates $$\begin{bmatrix} X&Y \end{bmatrix}^\top$$ via homography:

1 $$\begin{aligned} s \begin{bmatrix} u \\ v \\ 1 \end{bmatrix} = \textbf{H} \begin{bmatrix} X \\ Y \\ 1 \end{bmatrix}, \end{aligned}$$

where $\textbf{H} \in \mathbb {R}^{3\times 3}$ is the homography matrix. Decomposition with the camera intrinsics $\textbf{K}$ yields the translation $\textbf{t}$ and rotation matrix columns $\textbf{r}_1,\textbf{r}_2$, with $\textbf{r}_3 = \textbf{r}_1 \times \textbf{r}_2$. This provides drift-free global localization.

### Stage 2: IMU- and odometry-based tracking

Each robot publishes local odometry and IMU data. The payload pose is inferred through robot kinematics:

2 $$\begin{aligned} x_c&= x_i + l_i \cos (\theta _i + \phi _i), \end{aligned}$$

3 $$\begin{aligned} y_c&= y_i + l_i \sin (\theta _i + \phi _i), \end{aligned}$$

where $(x_i,y_i,\theta _i)$ is the robot pose, $l_i$ is the prismatic joint extension, and $\phi _i$ is the revolute joint angle. IMU feedback corrects short-term slip and encoder noise, ensuring reliable high-frequency updates.

### Stage 3: sensor fusion via Kalman filter

The available measurements are fused in a discrete Kalman filter to obtain a smooth, drift-minimized estimate. The state vector is

4 $$\begin{aligned} \textbf{x}_k = \begin{bmatrix} x_c&y_c&\theta _c \end{bmatrix}^\top , \quad \textbf{A} = \textbf{I}_3, \quad \textbf{C} = \textbf{I}_3, \end{aligned}$$

with standard recursion:

5 $$\begin{aligned} \hat{\textbf{x}}_{k|k-1}&= \textbf{A}\hat{\textbf{x}}_{k-1|k-1}, \quad \textbf{P}_{k|k-1} = \textbf{A}\textbf{P}_{k-1|k-1}\textbf{A}^\top + \textbf{Q}, \end{aligned}$$

6 $$\begin{aligned} \textbf{K}_k&= \textbf{P}_{k|k-1}\textbf{C}^\top (\textbf{C}\textbf{P}_{k|k-1}\textbf{C}^\top + \textbf{R})^{-1}, \end{aligned}$$

7 $$\begin{aligned} \hat{\textbf{x}}_{k|k}&= \hat{\textbf{x}}_{k|k-1} + \textbf{K}_k (\textbf{z}_k - \textbf{C}\hat{\textbf{x}}_{k|k-1}), \end{aligned}$$

where $\textbf{z}_k$ combines *camera + IMU + odometry* if the camera is available, or *IMU + odometry only* otherwise. This hierarchy guarantees real-time, resilient estimation for MPC feedback.

### ROS 2 integration

The pipeline is modularized into ROS 2 nodes:

- Camera node (optional): Publishes RGB images.
- Marker detection node: Computes global pose via homography.
- Encoder/IMU nodes: Publish local robot states.
- Workspace transformation node: Converts measurements to global frame.
- KF node: Fuses all data into the final object pose.
- MPC nodes: Subscribe to the fused estimate for control input generation.

## Decentralized nonlinear MPC

We formulate a *decentralized* nonlinear model predictive controller (NMPC) in which each robot $i \in \mathcal {I}:= \{1,2\}$ solves its own optimal control problem (OCP) using local measurements, local constraints, and a lightweight estimate of its peer. The controller must: (i) track the payload-center reference trajectory, (ii) maintain formation spacing and alignment, (iii) enforce state/input safety with hard and soft constraints, and (iv) combine offline meta-tuned weights with an online adaptive weighting mechanism. Real-time execution is performed using SQP-RTI with explicit Runge–Kutta (RK) integration and warm starts.

We consider discrete-time indices $k \in \{0{:}N\}$ with sampling time $T_s > 0$ and prediction horizon $N \in \mathbb {N}$. The cooperative transport task is defined by sampling a geometric path $\gamma (s)$ (parameterized by arc length *s*) to obtain reference states $g_k = [g_{x,k}, g_{y,k}, g_{\theta ,k}]^\top$. The payload center is denoted $q_k:= [x_{c,k}, y_{c,k}]^\top$. Inter-robot spacing is measured as $d_k^2:= (x_{i,k} - \hat{x}_{j,k})^2 + (y_{i,k} - \hat{y}_{j,k})^2$. Input increments are

8 $$\begin{aligned} \Delta \textbf{u}_{i,k} := \textbf{u}_{i,k} - \textbf{u}_{i,k-1}, \qquad \Delta \textbf{u}_{i,0} := \textbf{0}. \end{aligned}$$

Throughout this section, bold symbols denote vectors (e.g., $\textbf{x}_{i,k}$), while plain symbols denote scalar components (e.g., $x_{i,k}$). We use $\Vert z\Vert _W^2:= z^\top W z$ for a quadratic form and $\textrm{wrap}_{(-\pi ,\pi ]}(\cdot )$ to wrap angles to $(-\pi ,\pi ]$ (Table 1).

Notation and operators.

- $\textbf{x}_{i,k}$: 5D robot state; $\textbf{u}_{i,k}$: 2D control input.
- $\textrm{clip}(z,[a,b])$: projection of *z* onto [*a*, *b*].
- $$\textrm{wrap}_{(-\pi ,\pi ]}(\cdot )$$: angle normalization.
- $f_i(\textbf{x},\textbf{u})$: discrete-time dynamics.
- $h(\cdot )$: stacked inequality constraints.
- $W_k, W_N$: stage and terminal weight matrices.
- $r_\perp , r_\theta$: normalization constants for adaptive weighting.

**Table 1:** Key symbols, constants, and parameters used in “Decentralized nonlinear MPC”.

Symbol	Meaning
$T_s$	Sampling time
*N*	MPC prediction horizon
$\lambda$	Peer-state filtering gain
$\delta _k$	Communication delay (steps)
$\varepsilon _x(\cdot )$	Bound on peer-estimation error
$r_\perp , r_\theta$	Reference error scales
$k_{p,\chi }, k_{i,\chi }, k_{d,\chi }$	Log–PI+D gains ($\chi \in \{\perp ,\theta \}$)
$\alpha$	Log-weight smoothing factor
$w_{\min }, w_{\max }$	Log-weight box constraints
$v_{\max }, \omega _{\max }$	Velocity and angular-velocity limits
$a_{v,\max }, a_{\omega ,\max }$	Acceleration limits
$R_\Delta$	Input-increment penalty matrix
$d_{\min }, d_{\max }$	Formation spacing bounds
$\kappa _{\textrm{eff}}$	Effective curvature along reference
$\textbf{o}, r, c$	Obstacle center, radius, clearance

### Local states, inputs, and dynamics

State and input. Each robot *i* is modeled as a unicycle with extended velocity states:

9 $$\begin{aligned} \textbf{x}_{i,k} = \begin{bmatrix} x_{i,k} \\ y_{i,k} \\ \theta _{i,k} \\ v_{i,k} \\ \omega _{i,k} \end{bmatrix}, \quad \textbf{u}_{i,k} = \begin{bmatrix} a_{v,i,k} \\ a_{\omega ,i,k} \end{bmatrix}, \end{aligned}$$

where $a_{v,i,k}$ and $a_{\omega ,i,k}$ are the linear and angular accelerations.

Continuous-time model.

10 $$\begin{aligned} \dot{x}_i&= v_i \cos \theta _i,&\dot{y}_i&= v_i \sin \theta _i,&\dot{\theta }_i&= \omega _i, \nonumber \\ \dot{v}_i&= a_{v,i},&\dot{\omega }_i&= a_{\omega ,i}. \end{aligned}$$

Discrete-time RK integration. Using explicit RK with *s* stages and RK weights $b_r$:

11 $$\begin{aligned} \begin{aligned} \mathbf {{\textbf {x}}}_{i,k+1}&= f_i(\textbf{x}_{i,k}, \textbf{u}_{i,k}) \\&= \textbf{x}_{i,k} + T_s\sum _{r=1}^s b_r\, \boldsymbol{\phi }_r(\textbf{x}_{i,k}, \textbf{u}_{i,k}), \end{aligned} \end{aligned}$$

where $\boldsymbol{\phi }_r(\cdot )$ is the *r*th RK stage derivative.

### Peer estimate, payload center, and formation

Peer estimate. Each robot maintains a filtered estimate $\hat{x}_{j,k}$ of its peer:

12 $$\begin{aligned} \hat{x}_{j,k} \leftarrow (1-\lambda )\hat{x}_{j,k-1} + \lambda x^{\textrm{peer}}_{j,k-\delta _k}, \quad \Vert \tilde{x}_{j,k}\Vert \le \varepsilon _x(\delta _k), \end{aligned}$$

where $x^{\textrm{peer}}_{j,k-\delta _k}$ is the delayed received message.

Payload-center reconstruction.

13 $$\begin{aligned} x_{c,k}&\approx \tfrac{1}{2}(x_{i,k} + \hat{x}_{j,k}),&y_{c,k}&\approx \tfrac{1}{2}(y_{i,k} + \hat{y}_{j,k}), \nonumber \\ \theta _{c,k}&\approx \tfrac{1}{2}(\theta _{i,k} + \hat{\theta }_{j,k}). \end{aligned}$$

Formation spacing and alignment.

14 $$\begin{aligned} \begin{aligned} d_{\textrm{ref}}^2&= {\left\{ \begin{array}{ll} \tfrac{1}{4}(d_{\min }^2 + d_{\max }^2), & d_{\max } > d_{\min },\\ d_{\min }^2, & d_{\max } = d_{\min }, \end{array}\right. }\\ e_{\textrm{align},k}&:= \theta _{i,k} - \hat{\theta }_{j,k}. \end{aligned} \end{aligned}$$

### Frenet errors and reference sampling

Let $\Delta x_k:= x_{c,k}-g_{x,k}$, $\Delta y_k:= y_{c,k}-g_{y,k}$, and define shorthand trigonometric terms

$$(c_{\theta ,k}, s_{\theta ,k}) := (\cos g_{\theta ,k}, \sin g_{\theta ,k}).$$

Then the Frenet-frame errors are

15 $$\begin{aligned} \begin{aligned} e_{\parallel ,k}&= c_{\theta ,k}\Delta x_k + s_{\theta ,k}\Delta y_k,\\ e_{\perp ,k}&= -s_{\theta ,k}\Delta x_k + c_{\theta ,k}\Delta y_k,\\ e_{\theta ,k}&= \textrm{wrap}_{(-\pi ,\pi ]}\!\big (\theta _{c,k}-g_{\theta ,k}\big ). \end{aligned} \end{aligned}$$

Sampling uses arc-length steps $\Delta s \approx 0.15\,v_{\max }T_s$:

$$s_{k+1} = s_k + \Delta s, \quad g_k = [g_x(s_k),g_y(s_k),g_\theta (s_k)]^\top .$$

### Residuals, cost, and terminal terms

16 $$\begin{aligned} \quad \begin{aligned} \textbf{y}_{i,k}&= \begin{bmatrix} e_{\parallel ,k} \\ e_{\perp ,k} \\ e_{\theta ,k} \\ v_{i,k} \\ \omega _{i,k} \\ a_{v,i,k} \\ a_{\omega ,i,k} \\ (d_k^2 - d_{\textrm{ref}}^2) \\ e_{\textrm{align},k} \end{bmatrix} \quad , \textbf{y}_{i}^{N} = \begin{bmatrix} e_{\parallel ,N} \\ e_{\perp ,N} \\ e_{\theta ,N} \\ (d_N^2 - d_{\textrm{ref}}^2) \end{bmatrix}. \end{aligned} \end{aligned}$$

Weighting matrices.

17 $$\begin{aligned} \begin{aligned} W_k&= \textrm{diag}\!\big ( w_{\parallel ,k},w_{\perp ,k},w_{\theta ,k}, w_v,w_\omega ,w_{a,k},w_{a,k},w_d,w_{\textrm{align}} \big ),\\ W_N&= \textrm{diag}\!\big ( w_{\parallel }^{(e)}, w_{\perp }^{(e)}, w_{\theta }^{(e)}, w_{d}^{(e)} \big ). \end{aligned} \end{aligned}$$

Local cost.

18 $$\begin{aligned} \begin{aligned} J_i&= \sum _{k=0}^{N-1} \big ( \Vert \textbf{y}_{i,k}\Vert _{W_k}^2 + \Delta \textbf{u}_{i,k}^\top R_\Delta \Delta \textbf{u}_{i,k} \big ) + \Vert \textbf{y}_i^N\Vert _{W_N}^2. \end{aligned} \end{aligned}$$

### Optimization of weights: offline and online

Offline meta-tuning. We compute global priors $\phi$ by minimizing

19 $$\begin{aligned} \boxed { \begin{aligned} J_{\textrm{meta}}(\phi )&= \alpha \,\textrm{RMS}(e_\perp ) + \beta \,\textrm{RMS}(e_\theta ) \\&\quad + \gamma \,\mathbb {E}[\Vert \Delta \textbf{u}\Vert _2] + \delta \,\mathbb {E}[\textrm{slack}] + P_{\textrm{fail}}. \end{aligned}} \end{aligned}$$

The resulting $\phi ^\star$ initializes weights via a curvature-speed-aware map $(W_k)_0 = \mathcal {M}(\phi ^\star ; v, \kappa )$.

Online Log–PI+D adaptation. Normalized tracking signals:

$$\rho _{\perp ,k} = \frac{\bar{e}_{\perp ,k}}{r_\perp },\qquad \rho _{\theta ,k} = \frac{\bar{e}_{\theta ,k}}{r_\theta },$$

with finite differences

$$\dot{\rho }_{\chi ,k} = \frac{\rho _{\chi ,k}-\rho _{\chi ,k-1}}{T_s}, \quad \chi \in \{\perp ,\theta \}.$$

For each channel $\chi$:

20 $$\begin{aligned} \begin{aligned} I_{\chi ,k}&= \textrm{clip}\!\Big ( I_{\chi ,k-1} + k_{i,\chi }(\rho _{\chi ,k}-1),\, [I_{\min },I_{\max }] \Big ),\\ \ell _{\chi ,k}^{+}&= \textrm{clip}\!\Big ( \ell _{\chi ,k-1} + k_{p,\chi }(\rho _{\chi ,k}-1) + I_{\chi ,k} \\&\qquad \qquad + k_{d,\chi }\dot{\rho }_{\chi ,k},\, [\log w_{\min },\log w_{\max }] \Big ),\\ \ell _{\chi ,k}&= (1-\alpha )\ell _{\chi ,k-1} + \alpha \ell _{\chi ,k}^{+},\\ w_{\chi ,k}&= e^{\ell _{\chi ,k}}. \end{aligned} \end{aligned}$$

Shaping (speed, curvature, slack).

21 $$\begin{aligned} \begin{aligned} w_{\perp ,k}&= e^{\ell _{\perp ,k}} (1+k_v\bar{v}_k)(1+k_\kappa \kappa _{\textrm{eff},k})\,\xi _k,\\ w_{\theta ,k}&= e^{\ell _{\theta ,k}}(1+k_{\theta v}\bar{v}_k),\\ \xi _k&= \max \!\Big \{0,\, 1 - \eta _{\textrm{relax}} \dfrac{\kappa _{\textrm{eff},k}}{\kappa _{\max }} \dfrac{\bar{v}_k}{v_{\max }} \Big \},\\ w_{a,k}&= \max \!\big \{ 0.1\,w_{a0}, \; w_{a0}(1 - k_s\tanh (\eta \bar{s}_k)) \big \}. \end{aligned} \end{aligned}$$

### Constraints (per robot)

Each robot is subject to the constraints in Table 2.

**Table 2:** Per-robot constraints (compact summary).

Type	Constraint
Initial state	$\textbf{x}_{i,0} = \textbf{x}_i^{\textrm{meas}}$
State and input limits	$v_{\min }\!\le v_{i,k}\!\le v_{\max },\; \omega _{\min }\!\le \omega _{i,k}\!\le \omega _{\max }$;$|a_{v,i,k}|\!\le a_{v,\max },\; |a_{\omega ,i,k}|\!\le a_{\omega ,\max }$
Input increments	$\Vert \Delta \textbf{u}_{i,k}\Vert _\infty \le \Delta u_{\max }$
Formation spacing	$d_{\min }^2 \le d_k^2 \le d_{\max }^2$;$d_{\min }^2 - d_k^2 \le \sigma _{1,k},\; d_k^2 - d_{\max }^2 \le \sigma _{2,k}$
Obstacle clearance	$\Vert q_k - o\Vert _2^2 \ge (r + c + \Delta _c(q_k))^2$
Robust margins	$d_k^2 \ge d_{\min }^2 + \sigma _d(\varepsilon _x)$;$\Vert q_k - o\Vert _2 \ge r + c + \Delta _c(q_k) + \sigma _o(\varepsilon _x)$
Alignment (optional)	Penalize $e_{\textrm{align},k}$ in $\textbf{y}_{i,k}$

Slack variables $\boldsymbol{\sigma }_k:= [\sigma _{1,k},\sigma _{2,k},\dots ]^\top \ge 0$ appear in softened spacing and obstacle inequalities.

### Local OCP (per robot *i*)

Robust spacing and obstacle margins:

22 $$\begin{aligned} \begin{aligned} d_k^2&\ge d_{\min }^2 + \sigma _d(\varepsilon _x),\\ \Vert q_k - o\Vert _2&\ge r + c + \Delta _c(q_k) + \sigma _o(\varepsilon _x). \end{aligned} \end{aligned}$$

The local OCP at each sampling step is:

23 $$\begin{aligned} \begin{aligned} \min _{\{\textbf{x}_{i,k},\textbf{u}_{i,k},\boldsymbol{\sigma }_k\}}&\sum _{k=0}^{N-1} \Big ( \Vert \textbf{y}_{i,k}\Vert _{W_k}^2 + \Delta \textbf{u}_{i,k}^\top R_\Delta \Delta \textbf{u}_{i,k} + \rho ^\top \boldsymbol{\sigma }_k \Big ) \\&\quad + \Vert \textbf{y}_i^N\Vert _{W_N}^2 \\ \text {s.t.}\quad&\textbf{x}_{i,k+1} = f_i(\textbf{x}_{i,k},\textbf{u}_{i,k}),\\&h(\textbf{x}_{i,k},\textbf{u}_{i,k};\hat{x}_{j,k},p_k) + \boldsymbol{\sigma }_k \ge 0,\quad \boldsymbol{\sigma }_k \ge 0,\\&\textbf{x}_{i,0} = \textbf{x}_i^{\textrm{meas}},\qquad \Delta \textbf{u}_{i,k} = \textbf{u}_{i,k}-\textbf{u}_{i,k-1}. \end{aligned} \end{aligned}$$

### Communication trigger and robustness

Robot *i* broadcasts its state only when deviations exceed a communication threshold:

$$\max \!\{\,|e_{\perp ,k}|,\;|e_{\theta ,k}|,\; |d_k^2-d_{\textrm{ref}}^2|\,\} > \eta _{\textrm{tr}}.$$

Between broadcasts, feasibility is preserved by robust margins (22) under the estimation error bound $\Vert \tilde{x}_{j,k}\Vert \le \varepsilon _x$.

### Solver and execution

Each local OCP is solved using ACADOS with SQP-RTI, Gauss–Newton Hessians, explicit RK integration, and (optional) condensing. The per-stage solver parameter is

$$p_k = [g_{x,k},g_{y,k},g_{\theta ,k}]^\top .$$

Warm starts are created by shifting the previous optimal trajectories.

### Decentralized MPC algorithm

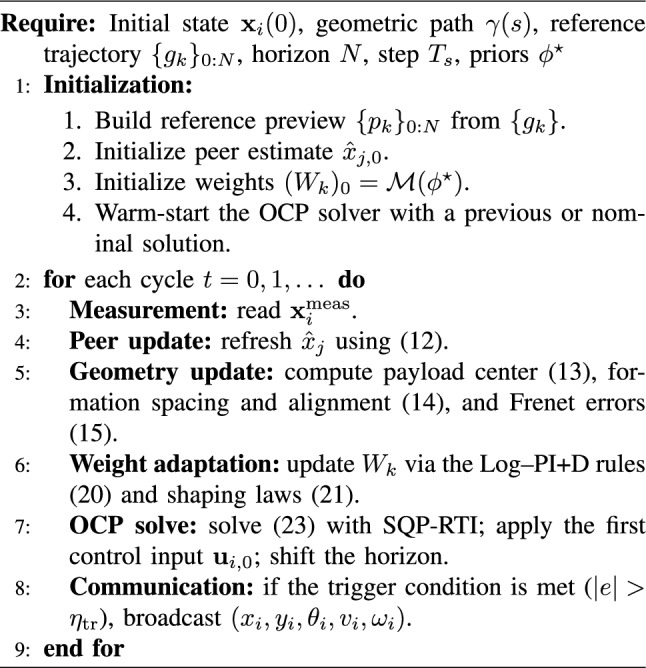



The method achieves: (i) improved tracking fidelity via adaptive weights that react to persistent errors; (ii) safe, smooth execution via robust margins, slack handling, and increment penalties; and (iii) scalability by solving OCPs locally with sparse, event-triggered communication.

## Experimental results

The decentralized NMPC of “Decentralized nonlinear MPC” was deployed on a dual-differential-drive testbed in a $3{\times }3$ m$^2$ arena. Each robot solved its local OCP (23) at 10 Hz with SQP-RTI and warm starts. The estimation layer combined visual measurements from an overhead camera with odometry and IMU data through an extended Kalman filter (EKF). Two phases of experiments were conducted: (i) a single-goal heading task using primarily camera-based localization with KF fusion, and (ii) multi-scenario trajectory tracking using odometry+IMU fused with KF as the primary localization source.

### Single goal-heading with camera fusion

**Fig. 5:**
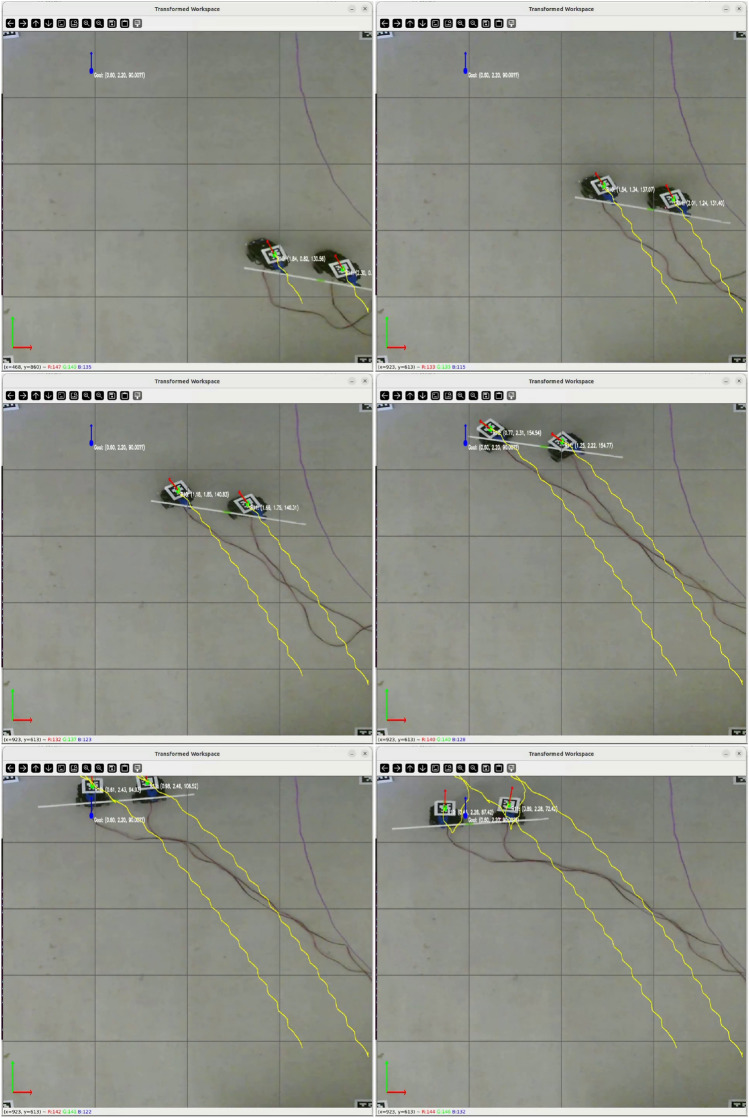
Physical point-to-point convergence using camera + KF fusion. Sequence (3 $\times$ 2 frames) shows smooth payload progression through two right-angle turns while maintaining formation.

We begin with the simplest baseline: convergence to a single goal point under camera-based localization fused with KF. The payload center $q_k$ was commanded to move from (0.3, 0.4) m to (2.5, 2.4) m with two right-angle turns. The KF fused visual position with velocity estimates, ensuring continuity across short vision dropouts. Figure 5 shows a 3 $\times$ 2 frame sequence extracted from the physical experiment video. The robots smoothly align to the reference, maintaining spacing and orientation alignment (14). Notably, during sharp turns the Log–PI+D update (20) raised $w_{\perp },w_{\theta }$, reducing lateral/heading errors without causing jerky inputs thanks to increment regularization (18).

### Trajectory tracking with odom+IMU fusion

Subsequent scenarios relied on EKF fusion of wheel odometry and IMU measurements, with the camera used only for ground-truth validation. Four representative paths were executed:

1. Circle of radius 1.2 m,
2. Line displacement across the arena,
3. Lemniscate (no obstacles), arc length $\approx 6.2$ m,
4. Lemniscate with obstacles, two cylindrical discs of radius 0.20 m.

**Fig. 6:**
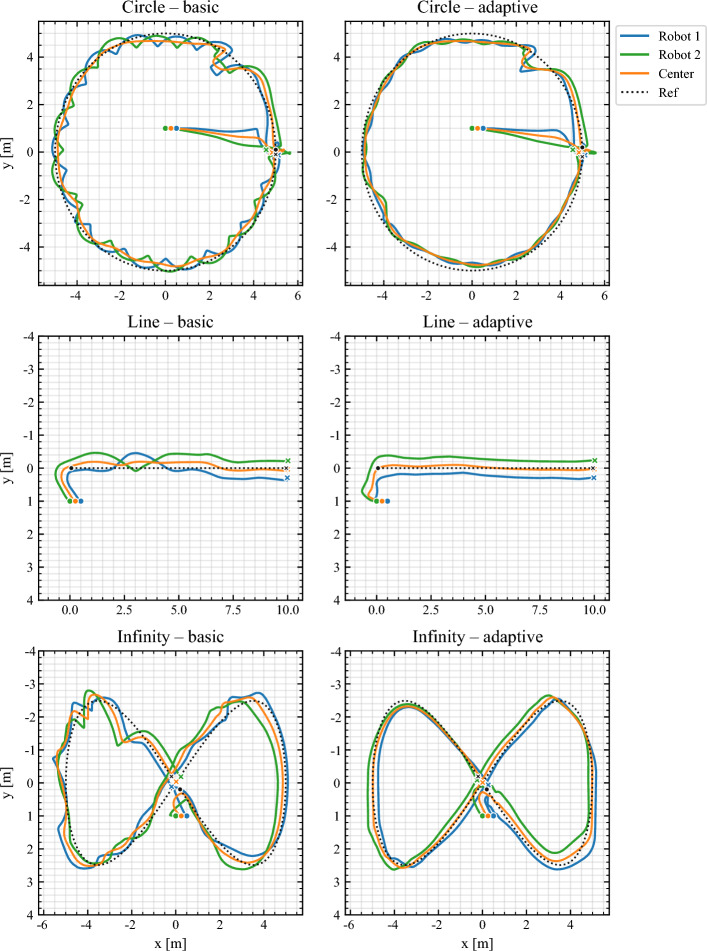
Representative trajectories (circle, line, lemniscate without obstacles). Adaptive NMPC suppresses curvature-induced drift while preserving smooth actuation.

**Fig. 7:**
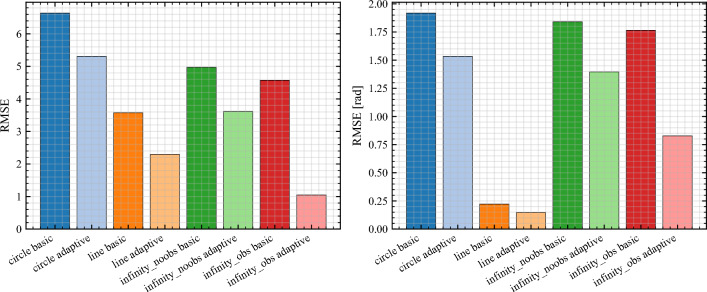
Aggregate RMSE across scenarios. Adaptive NMPC improves center tracking accuracy by 18–32% relative to fixed-weight baselines.

Figure 6 overlays robot and payload center trajectories for the circle, line, and obstacle-free lemniscate cases under both *basic* (fixed weights) and *adaptive* (Log–PI+D) controllers. Adaptive NMPC yields visibly tighter envelopes, particularly at curvature transitions. The aggregate RMSE comparison in Fig. 7 confirms 18–32% error reduction with adaptive weighting, consistent with the shaping law in (21) that scales weights with speed and effective curvature.

### Obstacle-aware lemniscate

**Fig. 8:**
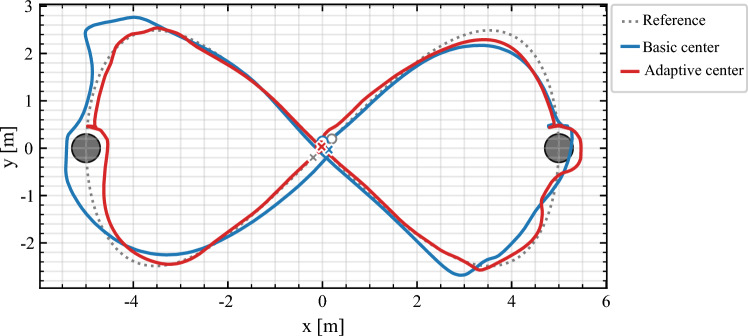
Obstacle-aware lemniscate. The *basic* controller (blue) exhibits lateral drift at high-curvature inflection points, reducing obstacle clearance. The *adaptive* controller (ref) tightens $w_{\perp },w_{\theta }$ exactly where curvature is high, keeping the payload center close to the reference (black). This results in both smaller lateral error and larger clearance margins.

The most demanding scenario combines curvature with obstacle avoidance. Figure 8 compares payload center trajectories against the reference. With basic NMPC, lateral drift accumulates near inflection points, occasionally narrowing clearance to the obstacles. The adaptive controller raises $w_{\perp },w_{\theta }$ where $\kappa _\textrm{eff}$ is large, aligning to the reference while maintaining positive margins.

**Fig. 9:**
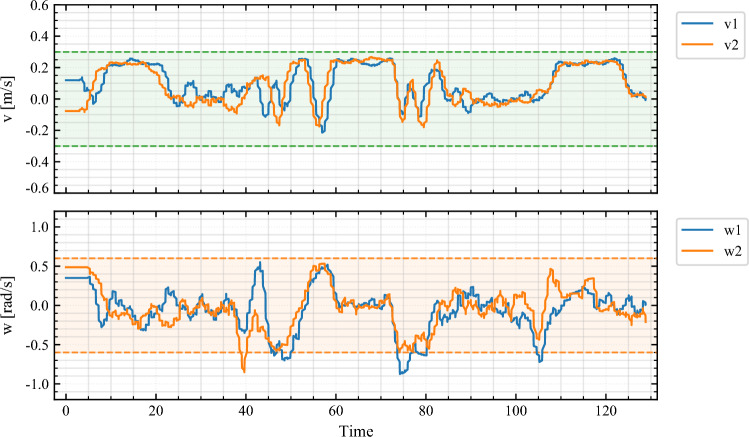
Adaptive NMPC input profiles during obstacle-aware lemniscate. Robot linear velocities $v_{1},v_{2}$ (top) and angular velocities $\omega _{1},\omega _{2}$ (bottom) remain smooth and bounded. Increment penalties reduce control rate change by $\sim$31%, ensuring jerk-free execution and actuator safety.

The corresponding input profiles are shown in Fig. 9. Both robots share velocity and turning effort smoothly, even during evasive peaks. Linear speeds remain below 0.28 m/s and angular rates within $\pm 1.5$ rad/s, well inside actuator limits. The increment penalty in (18) reduces inter-sample rate changes by $\sim$31%, evident from the smoother profiles of $\Delta v$ and $\Delta \omega$. This is crucial for hardware deployment, preventing wheel slip and actuator saturation.

### Error and constraint metrics

**Fig. 10:**
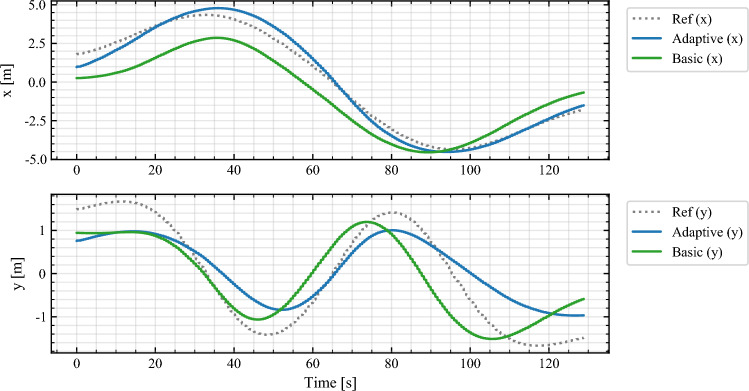
Time evolution of the payload center trajectory along the obstacle-aware lemniscate. The adaptive controller tracks both the *x*(*t*) and *y*(*t*) reference more closely than the basic fixed-weight NMPC, especially near high-curvature regions.

Figure 10 shows the time evolution of the payload center coordinates *x*(*t*) and *y*(*t*) relative to the reference path. These curves provide the requested trajectory-tracking behavior over time. The adaptive Log–PI+D weighting achieves noticeably tighter tracking than the fixed-weight NMPC, especially near curvature transitions where reference changes are fastest. This reflects the controller’s ability to increase $w_{\perp }$ and $w_{\theta }$ when tracking errors persist, while still preserving smooth, non-oscillatory inputs. The absence of abrupt deviations or overshoot in *x*(*t*) and *y*(*t*) indicates that the adaptive weights do not destabilize the system and remain compatible with real-time feasibility constraints.

### Solver timing and real-time feasibility

We benchmarked acados under $T_s{=}0.1$ s with horizons $N\in \{10,15,20,40,60,80,100,120,140\}$. With $N=15$, mean solve time is 9.7 ms, 95th percentile 13.9 ms, with zero deadline misses. At larger horizons, solve time increases roughly linearly with *N*, but deadlines remain satisfied up to $N=100$. At $N\ge 120$, occasional misses ($<1\%$) appear at curvature peaks, yet are absorbed by zero-order-hold without observable degradation (Table 3).

**Table 3:** Solver timing (Intel i7 NUC; single core pinned) across horizon lengths.

Horizon *N*	Mean (ms)	95th perc. (ms)	Deadline miss rate
10	6.8	9.5	0.0%
15	9.7	13.9	0.0%
20	13.8	19.6	0.0%
40	25.9	34.7	0.0%
60	38.6	52.4	0.0%
80	52.1	68.5	0.0%
100	65.3	86.1	0.0%
120	79.4	104.8	0.6%
140	93.7	121.5	0.9%

The experiments confirm the threefold advantage of the proposed scheme: (i) adaptive weighting improves reference fidelity by 18–32% without constraint violations; (ii) robust margins and slack handling ensure safety in obstacle-rich trajectories; (iii) increment penalties yield smooth actuation and faster solver convergence. Together, these validate the methodology of “Decentralized nonlinear MPC” on hardware, demonstrating that decentralized adaptive NMPC with KF fusion can deliver safe, accurate, and real-time multi-robot transport in constrained environments.

## Conclusion

We experimentally validated a decentralized nonlinear MPC framework for cooperative payload transport with two differential-drive robots coupled through revolute-prismatic joints. On hardware, the controller achieved sub-4 cm positional RMSE across point-to-point, curvilinear, and obstacle-rich tasks while strictly respecting inter-robot, clearance, and joint constraints. The ACADOS-based SQP-RTI solver met 10 Hz deadlines on embedded-class compute, and event-triggered communication reduced network load by $\sim$ 70% without loss of performance. These results confirm decentralized constrained MPC as a practical, real-time solution for cooperative transport and underline its capability for direct integration within robotic navigation stack layouts, where modularity, safety, and real-time feasibility are critical.

## Supplementary Information

Supplementary Information.

## Data Availability

No datasets were generated or analysed during the current study.
