# Supplementary material for: Real-time decentralized model predictive control for cooperative multi-robot object transport: experimental validation
Source: Sci Rep. 2026 Mar 22;16:9824. doi: 10.1038/s41598-026-41881-w (PMC13018580; doi:10.1038/s41598-026-41881-w)
Supplement: Supplementary file 1 — Supplementary Information. [file 41598_2026_41881_MOESM1_ESM.pdf]

Supplementary Files

Ibrahim Muhammed

October 2025

Supplementary Files

The supplementary materials include three ROS2 bag files collected during early Model Predictive Control (MPC) trials. Each file contains time-synchronized data from the depth camera, IMU, wheel encoders, odometry, and controller output topics.

- **Trial 1:** `MPC_1.bag` — baseline experiment with the initial MPC parameters.
- **Trial 2:** `MPC_2.bag` — repeated run with modified cost weights and prediction horizon.
- **Trial 3:** `MPC_3.bag` — experiment under external disturbance and dynamic conditions.

These datasets are available in the supplementary Google Drive folder at the following link: `Google Drive (MPC Trials Dataset)`.

The folder also contains a brief `plot.py` file that can help is plotting the robot position over time.
All topic list, and nodes can be accessed by replaying the bags using `ros2 bag play`. All files are provided to support reproducibility and further analysis.
